# E-cigarette use and associated factors among adolescents and young adults in northern Thailand: evidence from a population-based household survey

**DOI:** 10.3389/fpubh.2026.1813165

**Published:** 2026-03-25

**Authors:** Myo Zin Oo, Pintip Kaewkamthong, Tiwa Kaewserm, Kanittha Thaikla

**Affiliations:** 1 Research Institute for Health Sciences, Chiang Mai University, Chiang Mai, Thailand; 2 Faculty of Science and Technology, Phetchabun Rajabhat University, Phetchabun, Thailand; 3 Faculty of Agriculture and Industrial Technology, Phetchabun Rajabhat University, Phetchabun, Thailand

**Keywords:** adolescents, E-cigarettes, online exposure, population-based survey, substance use, Thailand, young adults

## Abstract

**Introduction:**

E-cigarette use is an emerging public health concern among adolescents and young adults, with limited population-based evidence on recent use and correlates in Thailand beyond school-based samples. Our study aimed to examine e-cigarette use and its associated factors among adolescents and young adults aged 15–29 years in Northern Thailand using data from a population-based household survey.

**Methods:**

We conducted a cross-sectional analysis of data from the 2024 population-based household survey on substance use in Northern Thailand. The analytic sample included 911 adolescents and young adults aged 15–29 years. Past-year e-cigarette use was the primary outcome. Explanatory variables encompassed sociodemographic characteristics, perceived accessibility, online information-seeking, perceived health harms, household stigma, and co-use of other substances. Descriptive statistics summarized prevalence, and logistic regression models estimated crude and adjusted associations, reporting odds ratios (ORs) and 95% confidence intervals (CIs). Statistical significance was defined as *p* < 0.05.

**Results:**

Overall, 11.5% of participants reported past-year e-cigarette use. In adjusted analyses, higher perceived accessibility (AOR = 3.18; 95% CI: 1.31, 7.69, *p* = 0.010), online information-seeking (AOR = 4.22; 95% CI: 2.36, 7.55, *p* < 0.001), and past-year alcohol use (AOR = 5.88; 95% CI: 2.83, 12.22, *p* < 0.001) were associated with higher odds of e-cigarette use. In contrast, perceiving e-cigarettes as harmful (AOR = 0.40; 95% CI: 0.23, 0.69, *p* = 0.001) and anticipating household stigma (AOR = 0.55; 95% CI: 0.32, 0.95, *p* = 0.031) were associated with lower odds of use.

**Conclusion:**

E-cigarette use among adolescents and young adults in Northern Thailand remains a public health concern shaped by accessibility, online exposure, alcohol co-use, and risk perceptions, underscoring the need for strengthened youth tobacco control, digital regulation, and integrated prevention.

## 1 Introduction

Electronic cigarette (e-cigarette) use has become a growing public health concern among adolescents and young adults, with recent surveillance data indicating high and increasing prevalence in this population (1). Early nicotine exposure during adolescence and young adulthood may have enduring neurodevelopmental effects that influence later substance use behaviors (2). Although often framed as harm-reduction tools for adult smokers, e-cigarette use among young people is associated with nicotine dependence and adverse respiratory and neurodevelopmental outcomes, with emerging evidence suggesting potential cardiovascular harms (3–6). The growing normalization of e-cigarette use within adolescent social environments, driven by peer dynamics, digital media content, and commercial marketing, may be facilitating higher levels of uptake among young people, with important implications for early nicotine initiation and longer-term health risks (7–9). This pattern highlights the importance of systematically characterizing the prevalence of e-cigarette use and its associated factors among adolescents to inform targeted public health responses.

In Thailand, the sale and importation of electronic cigarettes are prohibited under national legislation (10). Nevertheless, e-cigarettes continue to be accessible through informal markets and online platforms, contributing to ongoing youth exposure despite formal regulatory restrictions. Recent World Health Organization reporting documents rising e-cigarette use among Thai school-aged adolescents and highlights youth exposure to e-cigarette promotion via social media and influencer channels in the context of the sales ban (11). National school-based data further suggest that e-cigarette use among Thai youth is associated with modifiable factors, including limited awareness of harms and co-use of other substances (12). Together, these findings indicate a multi-level risk environment shaped by regulatory context, social exposure, and youth perceptions.

Despite growing policy attention and concern regarding youth e-cigarette use in Thailand, population-based evidence characterizing recent e-cigarette use and its correlates among adolescents and young adults remains limited. In particular, less is known about how individual characteristics intersect with exposure environments, online information and access pathways, health risk perceptions, household stigma, and co-use of other substances in shaping recent e-cigarette use within this age group. A more integrated understanding of these multiple domains is important for capturing the complexity of e-cigarette-related risk in contemporary youth environments and for identifying subgroups that may benefit from targeted prevention efforts. By adopting a population-based household survey approach, this study enables the simultaneous assessment of individual characteristics, exposure environments, online pathways, risk perceptions, household stigma, and co-use of other substances within a single analytical framework. Thus, our study aimed to examine e-cigarette use and its associated factors among adolescents and young adults aged 15–29 years in Northern Thailand using data from a population-based household survey.

## 2 Methods

### 2.1 Study design

Our study employed a cross-sectional analytical design drawing on data from the 2024 population-based household survey on substance use in Northern Thailand.

### 2.2 Participants and data source

Participants were recruited as part of the 2024 population-based household survey on substance use conducted across provinces in Northern Thailand between March and May 2024. The survey enumerated household members aged 12–65 years who met residency criteria in sampled households. For the present study, the analytic sample was restricted to adolescents and young adults aged 15–29 years with complete information on lifetime and past-year e-cigarette use. The final analytic sample comprised 911 participants, and no additional exclusion criteria were applied.

### 2.3 Sample size and sampling

The 2024 population-based household survey on substance use in Northern Thailand was implemented using a stratified multi-stage cluster sampling design to recruit community-dwelling residents aged 12–65 years. For sampling purposes, the region was stratified according to the Office of the Narcotics Control Board (ONCB) regional divisions, comprising ONCB Region 5 (upper northern provinces) and ONCB Region 6 (lower northern provinces). Sampling was conducted through five sequential stages. First, provinces were selected within each ONCB region using systematic sampling with probability proportional to the population aged 12–65 years. Second, subdistricts or local administrative areas were sampled within selected provinces using probability proportional to size. Third, villages and urban communities were selected within sampled subdistricts using probability proportional to size. Fourth, households were systematically sampled within selected communities based on local household listings and community maps. Finally, within each sampled household, eligible members aged 12–65 years were stratified by sex, and one male and one female respondent were randomly selected using a random number table, provided they were able to communicate effectively and consented to participate. Sex was recorded during the interview with response options of male, female, and other. A small number of respondents selected “other,” and no additional specification was provided.

**Figure 1:**
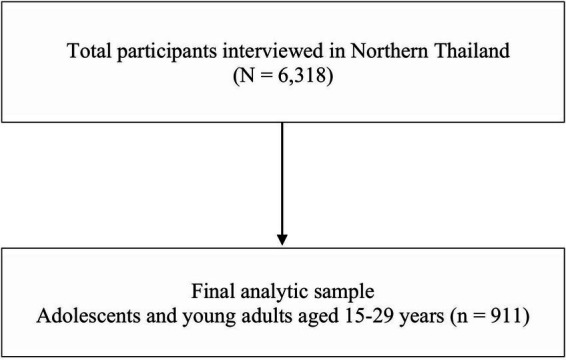
Study flow diagram. Among 6,318 participants interviewed in Northern Thailand, 911 were adolescents and young adults aged 15–29 years. All 911 participants had complete information on lifetime and past-year e-cigarette use and were included in the final analytic sample.

This sampling strategy yielded a total of 3,159 households and 6,318 individual respondents across Northern Thailand. For the present study, the analytic sample was restricted to adolescents and young adults aged 15–29 years, resulting in a final sample size of 911 participants (Figure 1). The multistage sampling approach was designed to ensure broad geographic coverage across urban and non-municipal areas of Northern Thailand, while preserving diversity across sociodemographic contexts.

### 2.4 Training and data collection process

Before field implementation, preparatory activities for the 2024 population-based household survey were undertaken through coordination meetings with the nationwide research network and participating academic institutions. Field teams received structured training on study objectives, questionnaire administration, household enumeration procedures, informed consent, and interview techniques, guided by standardized field operation and data coding manuals. Engagement with local leaders in each selected community facilitated access to study sites and supported the development of community maps and household listings.

Data collection in Northern Thailand was carried out by trained teams from the Research Institute for Health Sciences, Chiang Mai University (responsible for ONCB Region 5), and Phetchabun Rajabhat University (responsible for ONCB Region 6). Interviews were administered in sampled households in accordance with the multi-stage sampling protocol. Household rosters were compiled to identify eligible members, after which selected respondents provided informed consent and completed individual questionnaires. Data completeness, accuracy, and internal consistency were checked during fieldwork and subsequently verified during data entry and processing to support data quality assurance.

### 2.5 Measurements

Data were collected using standardized household and individual interview questionnaires developed as part of Thailand’s national household survey system on substance use, coordinated by the Academic Network on Substance Use (ACSAN) in collaboration with partner universities and the Office of the Narcotics Control Board. These survey instruments have been refined and implemented across multiple survey rounds since 2001 and are used for routine, periodic surveillance of substance use behaviors in Thailand. The standardized questionnaires covered multiple domains, including demographic and socioeconomic characteristics, physical and mental health status, quality of life, and substance use behaviors among household members aged 12–65 years. Substance use in the survey was assessed using experiential measures across multiple timeframes, in line with internationally accepted household survey practices.

For the present study, e-cigarette use was operationalized using the past-year (12-month) use measure from the survey. Variables were selected from the individual-level questionnaire and organized into two main domains: (1) explanatory variables, including sociodemographic characteristics, substance availability and exposure environment, online exposure and access pathways, perceived health harm and household stigma related to e-cigarette use, and co-use of other substances; and (2) the outcome variable, defined as past-year e-cigarette use.

### 2.6 Outcome variable

The primary outcome in our study was past-year e-cigarette use, derived from two questionnaire items. Respondents were first asked whether they had ever used e-cigarettes in their lifetime, and those reporting lifetime use were subsequently asked whether they had used e-cigarettes during the 12 months preceding the survey.

Responses were combined to classify participants into past-year users and non-past-year users. Past-year e-cigarette use was coded as 1 for respondents reporting lifetime use and any use in the previous 12 months, and coded as 0 for those reporting never use or lifetime use without past-year use. This operationalization aligns with standard practice in population-based substance use research and captures recent behavioral patterns that are more salient for public health relevance.

### 2.7 Explanatory variables

Explanatory variables in the present study were selected *a priori* based on the conceptual framework and existing literature and grouped into five domains: (1) sociodemographic characteristics (sex, age group, marital status, educational attainment, occupation, and monthly income); (2) availability and exposure environment (awareness of e-cigarettes, having seen e-cigarettes in real life, and perceived ability to obtain e-cigarettes); (3) online exposure and access pathways (searched/learned about e-cigarettes online, purchased e-cigarettes online); (4) risk perception and household stigma (perceived health harm of e-cigarettes, perceived household stigma of e-cigarette addiction); and (5) co-use of other substances in the past 12 months (cigarette smoking, alcohol use, and cannabis use). Age was categorized into 15–19 years (late adolescence), 20–24 years (early young adulthood), and 25–29 years (later young adulthood) to capture developmental differences in e-cigarette use. All explanatory variables were coded according to the original survey response categories and, where necessary, recategorized to ensure adequate cell sizes for analysis.

### 2.8 Data analysis

All analyses were conducted using STATA version 15. Descriptive statistics were used to summarize participants’ characteristics and the prevalence of past-year e-cigarette use, with results presented as frequencies and percentages. Bivariate associations between explanatory variables and past-year e-cigarette use were examined using Pearson’s χ^2^ tests, and the detailed results are presented in Supplementary Table. Variables demonstrating associations at *p* < 0.25 in bivariate analyses were considered for inclusion in multivariable models.

Binary logistic regression was employed to estimate unadjusted and adjusted associations between explanatory variables and past-year e-cigarette use. Adjusted odds ratios (AORs) with 95% confidence intervals (CIs) are reported. All covariates were entered simultaneously in the multivariable model based on theoretical relevance and prior empirical evidence to reduce confounding and omitted variable bias. The multivariable logistic regression model included age, sex, education, occupation, monthly income, perceived ability to obtain e-cigarettes, online exposure to e-cigarettes, perceived health harm, perceived household stigma, cigarette smoking, and alcohol use. Multicollinearity was assessed using variance inflation factors (VIFs), with no evidence of problematic collinearity (mean VIF = 3.09). Model adequacy was evaluated using likelihood ratio tests comparing nested models. Variables exhibiting complete separation (i.e., zero outcome events in one category), including the “other” sex category, awareness of e-cigarettes, and having seen e-cigarettes in real life, were not included in multivariable regression models because odds ratios could not be reliably estimated. Variables with very sparse cell counts (e.g., online purchase of e-cigarettes and cannabis use) were also excluded from the main multivariable model to avoid unstable estimates and model non-convergence.

The outcome variable (past-year e-cigarette use) had complete data for all participants (N = 911), whereas some explanatory variables had missing values; analyses therefore used available-case data, and sample sizes varied across models. Because sampling weights and full survey design variables (PSU and strata) were unavailable in the analytic dataset, analyses were conducted without weighting or complex survey adjustments, and findings are interpreted as reflecting associations within the surveyed sample. Statistical significance was defined as *p* < 0.05.

### 2.9 Ethics statement

The 2024 population-based household survey on substance use in Northern Thailand was approved by the Human Research Ethics Committee of Vongchavalitkul University (Approval No. 91/2567; approved on 13 March 2024). All participants provided informed consent prior to participation. Given the sensitive nature of substance use behaviors, strict confidentiality procedures were implemented, including the non-collection of personal identifiers, conducting interviews in private settings, and allowing self-administration of sensitive items when appropriate. The present analysis used de-identified data from the 2024 population-based household survey conducted in Northern Thailand.

## 3 Results

Table 1 summarizes the sociodemographic characteristics of the respondents. The sample (N = 911) was predominantly aged 25–29 years (37.2%), with a near-equal sex distribution (50.8% male, 49.0% female). Most participants had at least upper secondary education (66.9%), were never married (71.0%), and were students (41.5%); nearly half (46.2%) reported a monthly income of ≤5,000 Thai baht (THB) [US$1 = 35.29 THB, period-average exchange rate for 2024; World Bank (13)].

**Table 1:** Sociodemographic characteristics of the respondents (analytic sample, *N* = 911).

Variables	Number (N)	Percentage (%)
Sociodemographic characteristics		
Age		
15–19	310	34.0
20–24	262	28.8
25–29	339	37.2
Sex		
Male	463	50.8
Female	446	49.0
Other	2	0.2
Education (*n* = 907)		
Primary education or less	62	6.8
Lower secondary	238	26.3
Upper secondary	306	33.7
Post-secondary	301	33.2
Marital status		
Never married	647	71.0
In a relationship	258	28.3
Previously married	6	0.7
Occupation (*n* = 910)		
Not in labor force	71	7.8
Student	378	41.5
Employed (formal sector)	121	13.3
Self-employed/informal work	122	13.4
Manual labor/agriculture	218	24.0
Average monthly income (in Thai baht)^ǂ^ (*n* = 809)		
≤5,000	374	46.2
5,001–10,000	227	28.1
>10,000	208	25.7
Range (Min–Max)	500–69,000	

Values are presented as number (percentage). Percentages are calculated within each variable. Sample sizes vary due to missing data. ^ǂ^All monetary values are reported in Thai baht (THB); the 2024 period-average exchange rate was US$1 = 35.29 THB (World Bank) (13).

Table 2 summarizes availability, exposure, perceptions, and co-use of substances. Awareness and real-life exposure to e-cigarettes were high (91.0 and 91.4%), and most participants perceived e-cigarettes as easy to obtain (75.7%). Online engagement was limited (14.6% searched/learned online, 17.3% of these purchased online). Most perceived e-cigarettes as harmful (74.0%) and anticipated household stigma (59.8%), while alcohol use was common (49.9%), with lower prevalence of cigarette smoking (12.4%) and cannabis use (2.3%).

**Table 2:** Availability, exposure, perceptions, and co-use of substances (analytic sample, *N* = 911).

Variables	Number (N)	Percentage (%)
Availability and exposure		
Awareness of e-cigarettes		
No	82	9.0
Yes	829	91.0
Seen e-cigarettes in real life (*n* = 829)		
No	71	8.6
Yes	758	91.4
Perceived ability to obtain e-cigarettes (*n* = 773)		
No	188	24.3
Yes	585	75.7
Online exposure and access		
Searched/learned about e-cigarettes online		
No	778	85.4
Yes	133	14.6
Purchased e-cigarettes online (*n* = 133)		
No	110	82.7
Yes	23	17.3
Risk perception and stigma		
Perceived health harm of e-cigarettes (*n* = 910)		
No perceived harm	237	26.0
Perceived harm	673	74.0
Perceived household stigma of e-cigarette addiction (*n* = 910)		
Not stigmatizing	366	40.2
Stigmatizing	544	59.8
Co-use of other substances		
Cigarette smoking (past 12 months)		
No	798	87.6
Yes	113	12.4
Alcohol use (past 12 months)		
No	456	50.1
Yes	455	49.9
Cannabis use (past 12 months)		
No	890	97.7
Yes	21	2.3

Values are presented as number (percentage). Percentages are calculated within each variable. Sample sizes vary due to missing data and skip patterns in the original questionnaire.

Table 3 presents the prevalence of past-year e-cigarette use among adolescents and young adults. Overall, 11.5% of respondents reported using e-cigarettes in the past 12 months, while the majority (88.5%) reported no past-year use.

**Table 3:** Prevalence of past-year e-cigarette use (analytic sample, *N* = 911).

Past-year e-cigarette use	Number (N)	Percentage (%)
No	806	88.5
Yes	105	11.5

Values are presented as number (percentage).

Table 4 presents factors associated with past-year e-cigarette use. In the multivariable analysis, participants aged 25–29 years had lower odds of past-year use than those aged 15–19 years (AOR = 0.26; 95% CI: 0.09, 0.71, *p* = 0.008), and females were less likely to use e-cigarettes than males (AOR = 0.43; 95% CI: 0.24, 0.81, *p* = 0.008). Greater environmental and online exposure were associated with higher odds of use, including perceived ability to obtain e-cigarettes (AOR = 3.18; 95% CI: 1.31, 7.69, *p* = 0.010) and having searched for or learned about e-cigarettes online (AOR = 4.22; 95% CI: 2.36, 7.55, *p <* 0.001). In contrast, perceiving e-cigarettes as harmful (AOR = 0.40; 95% CI: 0.23, 0.69, *p* = 0.001) and anticipating household stigma (AOR = 0.55; 95% CI: 0.32, 0.95, *p* = 0.031) were associated with lower odds of use. Alcohol use remained a strong correlate (AOR = 5.88; 95% CI: 2.83, 12.22, *p* < 0.001), whereas cigarette smoking was not significant after adjustment.

**Table 4:** Factors associated with past-year e-cigarette use.

Variables	Past-year e-cigarette use			
	Crude OR (95% CI)	*p*-value	Adjusted OR (95% CI)	*p*-value
Age		0.001*		0.025*
15–19	1 (ref)		1 (ref)	
20–24	1.18 (0.74, 1.89)	0.478	0.55 (0.25, 1.25)	0.153
25–29	0.44 (0.26, 0.76)	0.003*	0.26 (0.09, 0.71)	0.008*
Sex		<0.001**		0.008*
Male	1 (ref)		1 (ref)	
Female	0.25 (0.16, 0.41)	<0.001**	0.43 (0.24, 0.81)	0.008*
Other	Not estimable^ǂ^	–	Not estimable^ǂ^	–
Education		0.096		0.396
Primary education or less	1 (ref)		1 (ref)	
Lower secondary	1.45 (0.61, 3.42)	0.401	2.11 (0.65, 6.79)	0.212
Upper secondary	0.95 (0.40, 2.26)	0.907	1.29 (0.39, 4.22)	0.664
Post-secondary	0.74 (0.31, 1.80)	0.510	1.21 (0.35, 4.16)	0.766
Marital status		0.399		–
Never married	1 (ref)		1 (ref)	
In a relationship	0.73 (0.45, 1.18)	0.194	–	–
Previously married	1.42 (0.16, 12.29)	0.752	–	–
Occupation		0.002*		0.726
Not in labor force	1 (ref)		1 (ref)	
Student	0.72 (0.34, 1.52)	0.391	0.63 (0.15, 2.63)	0.526
Employed (formal sector)	0.37 (0.14, 1.03)	0.058	0.75 (0.14, 4.07)	0.741
Self-employed/informal work	0.43 (0.16, 1.14)	0.090	0.90 (0.18, 4.58)	0.903
Manual labor/agriculture	1.37 (0.65, 2.91)	0.411	1.13 (0.27, 4.76)	0.868
Average monthly income (in Thai baht) (*n* = 809)		0.032*		0.524
≤5,000	1 (ref)		1 (ref)	
5,001–10,000	1.16 (0.72, 1.86)	0.554	1.34 (0.62, 2.91)	0.459
>10,000	0.49 (0.26, 0.91)	0.024*	0.86 (0.32, 2.28)	0.757
Awareness of e-cigarettes		–		–
No	1 (ref)		1 (ref)	
Yes	Not estimable^ǂ^		Not estimable^ǂ^	
Seen e-cigarettes in real life				
No	1 (ref)	–	1 (ref)	–
Yes	Not estimable^ǂ^		Not estimable^ǂ^	
Perceived ability to obtain e-cigarettes		<0.001**		0.010*
No	1 (ref)		1 (ref)	
Yes	5.20 (2.37, 11.41)		3.18 (1.31, 7.69)	
Searched/learned about e-cigarettes online		<0.001**		<0.001**
No	1 (ref)		1 (ref)	
Yes	7.14 (4.58, 11.15)		4.22 (2.36, 7.55)	
Purchased e-cigarettes online^†^		<0.001**		–
No	1 (ref)		1 (ref)	
Yes	19.52 (5.39, 70.72)		–	
Perceived health harm of e-cigarettes		<0.001**		0.001*
No perceived harm	1 (ref)		1 (ref)	
Perceived harm	0.33 (0.22, 0.50)		0.40 (0.23, 0.69)	
Perceived household stigma of e-cigarette addiction		<0.001**		0.031*
Not stigmatizing	1 (ref)		1 (ref)	
Stigmatizing	0.38 (0.25, 0.58)		0.55 (0.32, 0.95)	
Cigarette smoking (past 12 months)		<0.001**		0.103
No	1 (ref)		1 (ref)	
Yes	4.94 (3.10, 7.87)		1.74 (0.89, 3.37)	
Alcohol use (past 12 months)		<0.001**		<0.001**
No	1 (ref)		1 (ref)	
Yes	9.51 (5.13, 17.62)		5.88 (2.83, 12.22)	
Cannabis use (past 12 months)^†^		<0.001**		–
No	1 (ref)		1 (ref)	
Yes	11.43 (4.69, 27.84)		–	

**Highly statistically significance: *p* < 0.001, * Statistically significance at *p* < 0.05, ^ǂ^Odds ratios could not be estimated due to complete separation (zero outcome events in one category) ^†^Variable excluded from the multivariable model due to sparse cell counts and unstable estimates, Model fit statistics: likelihood-ratio *χ*^2^ = 166.96 (*p* < 0.001); pseudo *R*^2^ = 0.31.

## 4 Discussion

Based on a population-based household survey of 911 adolescents and young adults aged 15–29 years in Northern Thailand, our study provides new evidence on the prevalence and associated factors of past-year e-cigarette use. Although the survey was designed to assess a wide range of substance use behaviors, the present analysis focused on e-cigarette use given its growing public health relevance among adolescents and young adults in Thailand.

We found that 11.5% of respondents reported using e-cigarettes in the past 12 months, while the majority (88.5%) reported no past-year use. This prevalence indicates that e-cigarette use is a notable and emerging public health concern among adolescents and young adults in Northern Thailand, despite the formal prohibition on the sale and importation of these products; a prevalence exceeding one in 10 suggests that e-cigarette use has moved beyond marginal experimentation within youth social environments. Our observed prevalence is higher than that reported in school- and city-based surveys of Thai adolescents (12, 14) but lower than estimates among Thai university students (15) and online adult samples (16), reflecting differences in age groups, study settings, and recall periods. Internationally, our estimate is comparable to pooled global youth prevalence (17), higher than that reported among young adults in Vietnam (18), and lower than that reported among high school and university students in Indonesia (19), highlighting cross-national variation in e-cigarette use across Southeast Asia. Considering these national and international comparisons, e-cigarette use among adolescents and young adults warrants prioritization within youth-focused tobacco control and prevention agendas in Thailand.

Age was independently associated with past-year e-cigarette use, with participants aged 25–29 years having significantly lower odds than those aged 15–19 years (AOR = 0.26; 95% CI: 0.09, 0.71; *p* = 0.008). These findings suggest that recent e-cigarette use was more concentrated among adolescents than among older young adults in this population. In contrast to Thai school-based data showing increasing e-cigarette use across early adolescence (12), our findings indicate lower use in later young adulthood, highlighting age-related variation in e-cigarette use across developmental stages and aligning with longitudinal evidence of dynamic e-cigarette use trajectories across adolescence and early adulthood (20). These age-related patterns suggest that prevention and early intervention efforts should prioritize adolescents, particularly during late adolescence, as a key window for e-cigarette use prevention in Northern Thailand. A clear sex difference was observed in past-year e-cigarette use, with females showing significantly lower odds of use than males (AOR = 0.43; 95% CI: 0.24, 0.81; *p* = 0.008). Our observed male predominance in e-cigarette use aligns with evidence from Vietnam showing significantly higher odds of use among male young adults compared with females (18). These findings suggest that prevention strategies should include gender-responsive approaches that specifically address higher e-cigarette use among males within youth health promotion and tobacco control programs.

Respondents who perceived e-cigarettes as easy to obtain had significantly higher odds of past-year use (AOR = 3.18; 95% CI: 1.31, 7.69; *p* = 0.010), indicating a close link between perceived accessibility and recent e-cigarette use among adolescents and young adults in Northern Thailand. Similar patterns have been observed in school-based studies from the United States, where adolescents commonly report easy access to e-cigarette products and higher use (21). This pattern highlights the importance of strengthening youth-focused tobacco control efforts that prioritize reducing perceived and actual accessibility of e-cigarettes through effective enforcement and age-appropriate prevention strategies. Participants who searched for or learned about e-cigarettes online had higher odds of past-year use (AOR = 4.22; 95% CI: 2.36, 7.55; *p* < 0.001). Bivariable analyses also indicated higher odds among those who reported purchasing e-cigarettes online (AOR = 19.52; 95% CI: 5.39, 70.72; *p* < 0.001) although this estimate was excluded from multivariable models due to sparse data. Together, these findings highlight the relevance of online environments for e-cigarette-related information-seeking and access, pointing to the need to strengthen regulation and monitoring of online e-cigarette marketing and sales accessible to adolescents and young adults.

Perceiving e-cigarettes as harmful was associated with lower odds of past-year use (AOR = 0.40; 95% CI: 0.23, 0.69; *p* = 0.001). This pattern aligns with evidence from Australia indicating that a substantial proportion of adolescents and young adults perceive e-cigarettes as harmful to health (22), highlighting the relevance of harm perceptions for e-cigarette-related behaviors. Overall, this supports the inclusion of risk communication strategies that address misperceptions about the harm of e-cigarettes within youth-focused prevention programs. Participants who perceived household stigma toward e-cigarette addiction had lower odds of past-year e-cigarette use (AOR = 0.55; 95% CI: 0.32, 0.95; *p* = 0.031), consistent with longitudinal evidence from the United States showing that strict home rules are associated with lower subsequent e-cigarette use among adolescents (23). These findings support the inclusion of family- and caregiver-focused components within youth tobacco prevention strategies.

Past-year alcohol use was strongly associated with past-year e-cigarette use (AOR = 5.88; 95% CI: 2.83, 12.22; *p* < 0.001), consistent with evidence from a recent systematic review and meta-analysis showing that alcohol use and binge drinking are associated with higher odds of e-cigarette use (24). These findings support integrated prevention approaches that address the co-use of alcohol and e-cigarettes among adolescents and young adults. Cigarette smoking and cannabis use were associated with e-cigarette use in bivariable analyses; however, the association for cigarette smoking was attenuated and no longer statistically significant after adjustment, and cannabis use could not be robustly assessed in adjusted models due to sparse data, indicating clustering of substance use behaviors without clear independent effects in multivariable analyses.

### 4.1 Limitation and strengths

Our study has certain limitations. Because of the cross-sectional design, causal and temporal relationships between exposures and past-year e-cigarette use cannot be established; longitudinal studies are needed to clarify directionality. Self-reported measures may be subject to recall and social desirability bias, with possible underreporting in restrictive legal contexts; future studies could complement self-reports with objective indicators (e.g., biochemical markers), where feasible. The absence of sampling weights and full survey design variables precluded adjustment for complex survey design, potentially limiting population representativeness and variance estimation. Some exposures were excluded from adjusted models due to sparse data or complete separation, limiting power and warranting cautious interpretation of their independent associations. While the binary past-year indicator is appropriate for prevalence estimation, more granular measures of frequency and intensity are needed to capture heterogeneity in e-cigarette use behaviors. Findings may not generalize beyond adolescents and young adults in private households, excluding more marginalized or mobile youth populations. Finally, the small number of past-year users limited power for some subgroup analyses, resulting in wider confidence intervals and cautious interpretation of certain estimates.

Our study has several notable strengths. First, it draws on a large population-based household survey with multi-stage cluster sampling, providing community-level evidence on e-cigarette use beyond school- or institution-based samples and capturing adolescents and young adults both in and out of formal education. Second, the use of recent (2024) survey data enhances the policy relevance of the findings in a rapidly evolving e-cigarette landscape in Thailand. Third, the study adopts an integrated analytical framework that simultaneously examines sociodemographic characteristics, exposure environments, online information and access pathways, risk perceptions, household stigma, and co-use of other substances, offering a more comprehensive understanding of the multi-level correlates of youth e-cigarette use. Fourth, the focus on past-year e-cigarette use provides a public health-relevant indicator of recent behavior beyond lifetime experimentation and short-term fluctuations. Finally, rigorous analytic procedures, including multivariable modeling, assessment of multicollinearity, and transparent reporting of modeling constraints (e.g., sparse data and complete separation), strengthen the credibility and interpretability of the findings.

### 4.2 Public health implications and recommendations

E-cigarette use among adolescents and young adults in Northern Thailand represents a meaningful public health concern despite regulatory bans, highlighting gaps in enforcement and the growing influence of online environments. Prevention strategies should prioritize adolescents and males, strengthen monitoring of informal and digital access pathways, and integrate e-cigarette prevention within broader youth substance use programs. Risk communication addressing misperceptions of harm and family-engaged approaches may offer protective leverage, while continued population-based surveillance is needed to guide responsive tobacco control policies.

## 5 Conclusion

Our population-based study provides recent evidence that e-cigarette use is a non-trivial and emerging public health concern among adolescents and young adults in Northern Thailand, despite national prohibitions on sales and importation. Use was more common among adolescents and males and was associated with greater accessibility, online information-seeking, alcohol use, and lower perceived harm and household stigma, reflecting multi-level drivers of youth e-cigarette use in restrictive regulatory contexts. These findings highlight the need for strengthened youth-focused tobacco control strategies that address informal and digital access pathways, integrate e-cigarette prevention within broader substance use prevention efforts, and reinforce accurate risk communication. Continued population-based surveillance and targeted prevention during adolescence are essential to mitigate early nicotine initiation and its longer-term public health consequences in Thailand.

## Data Availability

The data analyzed in this study is subject to the following licenses/restrictions: De-identified data may be made available from the corresponding author upon reasonable request and subject to approval in accordance with ethical and data governance requirements. Requests to access these datasets should be directed to kthaikla@gmail.com.
