# Supplementary material for: E-cigarette use and associated factors among adolescents and young adults in northern Thailand: evidence from a population-based household survey
Source: Front Public Health. 2026 Mar 25;14:1813165. doi: 10.3389/fpubh.2026.1813165 (PMC13057327; doi:10.3389/fpubh.2026.1813165)
Supplement: Supplementary file 1 [file Table_1.docx]

**Supplementary table** Bivariate (Chi-square) associations between independent variables and past-year e-cigarette use (analytic sample, N = 911)

| **Variables** | **Past-year e-cigarette use** | | ***p* value** |
| --- | --- | --- | --- |
|  | **No** | **Yes** |  |
|  | **N (%)** | **N (%)** |  |
| **Sociodemographic characteristics** |  |  |  |
| **Age** |  |  | 0.001* |
| 15-19 | 268 (86.4) | 42 (13.6) |  |
| 20-24 | 221 (84.4) | 41 (15.6) |  |
| 25-29 | 317 (93.5) | 22 (6.5) |  |
| **Sex** |  |  | <0.001** |
| Male | 381 (82.3) | 82 (17.7) |  |
| Female | 423 (94.8) | 23 (5.2) |  |
| Other | 2 (100.0) | 0 (0.0) |  |
| **Education (n=907)** |  |  | 0.091 |
| Primary education or less | 55 (88.7) | 7 (11.3) |  |
| Lower secondary | 201 (84.5) | 37 (15.5) |  |
| Upper secondary | 273 (89.2) | 33 (10.8) |  |
| Post-secondary | 275 (91.4) | 26 (8.6) |  |
| **Marital status** |  |  | 0.396 |
| Never married | 567 (87.6) | 80 (12.4) |  |
| In a relationship | 234 (90.7) | 24 (9.3) |  |
| Previously married | 5 (83.3) | 1 (16.7) |  |
| **Occupation (n=910)** |  |  | 0.001* |
| Not in labor force | 61 (85.9) | 10 (14.1) |  |
| Student | 338 (89.4) | 40 (10.6) |  |
| Employed (formal sector) | 114 (94.2) | 7 (5.8) |  |
| Self-employed/ informal work | 114 (93.4) | 8 (6.6) |  |
| Manual labor/ agriculture | 178 (81.6) | 40 (18.4) |  |
| **Average monthly income (in Thai baht)^ǂ^ (n=809)** |  |  | 0.028* |
| ≤ 5000 | 326 (87.2) | 48 (12.8) |  |
| 5001-10000 | 194 (85.5) | 33 (14.5) |  |
| > 10000 | 194 (93.3) | 14 (6.7) |  |
| **Availability & exposure** |  |  |  |
| **Awareness of e-cigarettes** |  |  | 0.001* |
| No | 82 (100) | 0 (0.0) |  |
| Yes | 724 (87.3) | 105 (12.7) |  |
| **Seen e-cigarettes in real life (n=829)** |  |  | 0.001* |
| No | 71 (100.0) | 0 (0.0) |  |
| Yes | 653 (86.2) | 105 (13.8) |  |
| **Perceived ability to obtain e-cigarettes (n=773)** |  |  | <0.001** |
| No | 181 (96.3) | 7 (3.7) |  |
| Yes | 487 (83.3) | 98 (16.7) |  |
| **Online exposure & access** |  |  |  |
| **Searched/learned about e-cigarettes online** |  |  | <0.001** |
| No | 721 (92.7) | 57 (7.3) |  |
| Yes | 85 (63.9) | 48 (36.1) |  |
| **Purchased e-cigarettes online** |  |  | <0.001** |
| No | 82 (74.5) | 28 (25.5) |  |
| Yes | 3 (13.0) | 20 (87.0) |  |
| **Risk perception & stigma** |  |  |  |
| **Perceived health harm of e-cigarettes** |  |  | <0.001** |
| No perceived harm | 187 (78.9) | 50 (21.1) |  |
| Perceived harm | 618 (91.8) | 55 (8.2) |  |
| **Perceived household stigma of e-cigarette addiction** |  |  | <0.001** |
| Not stigmatizing | 302 (82.5) | 64 (17.5) |  |
| Stigmatizing | 503 (92.5) | 41 (7.5) |  |
| **Co-use of other substances** |  |  |  |
| **Cigarette smoking (past 12 months)** |  |  | <0.001** |
| No | 729 (91.4) | 69 (8.6) |  |
| Yes | 77 (68.1) | 36 (31.9) |  |
| **Alcohol use (past 12 months)** |  |  | <0.001** |
| No | 444 (97.4) | 12 (2.6) |  |
| Yes | 362 (79.6) | 93 (20.4) |  |
| **Cannabis use (past 12 months)** |  |  | <0.001** |
| No | 797 (89.5) | 93 (10.5) |  |
| Yes | 9 (42.9) | 12 (57.1) |  |

**Highly statistically significance: *p* < 0.001, * Statistically significance at *p* <0.05

*P* values were derived from Pearson’s χ² tests, Values are presented as number (percentage), Due to missing data, sample sizes vary across variables as indicated, Cells with zero counts are shown as 0 (0.0); χ² results involving sparse cells should be interpreted with caution, ^ǂ^All monetary values are reported in Thai baht (THB); the 2024 period-average exchange rate was US$1 = 35.29 THB (World Bank) [13].
